# What's the gap? A possible strategy for advancing theory, and an appeal for experimental structure data to drive that advance†

**DOI:** 10.1039/d0ra07496a

**Published:** 2020-10-06

**Authors:** Karl Sohlberg, Michael E. Foster

**Affiliations:** Department of Chemistry, Drexel University Philadelphia PA 19104 USA sohlbergk@drexel.edu; Department of Materials Science & Engineering, Drexel University Philadelphia PA 19104 USA

## Abstract

There is substantial demand for theoretical/computational tools that can produce correct predictions of the geometric structure and band gap to accelerate the design and screening of new materials with desirable electronic properties. DFT-based methods exist that reliably predict electronic structure given the correct geometry. Similarly, when good spectroscopic data are available, these same methods may, in principle, be used as input to the inverse problem of generating a good structural model. The same is generally true for gas-phase systems, for which the choice of method is different, but factors that guide its selection are known. Despite these successes, there are shortcomings associated with DFT for the prediction of materials' electronic structure. The present paper offers a perspective on these shortcomings. Fundamentally, the shortcomings associated with DFT stem from a lack of knowledge of the exact functional form of the exchange–correlation functional. Inaccuracies therefore arise from using an approximate functional. These inaccuracies can be reduced by judicious selection of the approximate functional. Other apparent shortcomings present due to misuse or improper application of the method. One of the most significant difficulties is the lack of a robust method for predicting electronic and geometric structure when only qualitative (connectivity) information is available about the system/material. Herein, some actual shortcomings of DFT are distinguished from merely common improper applications of the method. The role of the exchange functional in the predicted relationship between geometric structure and band gap is then explored, using fullerene, 2D polymorphs of elemental phosphorus and polyacetylene as case studies. The results suggest a potentially fruitful avenue of investigation by which some of the true shortcomings might be overcome, and serve as the basis for an appeal for high-accuracy experimental structure data to drive advances in theory.

## Abstract 2

Strong correlation between electronic structure and geometry might be capitalized upon to tune the DFT functional.

## Graphical abstract

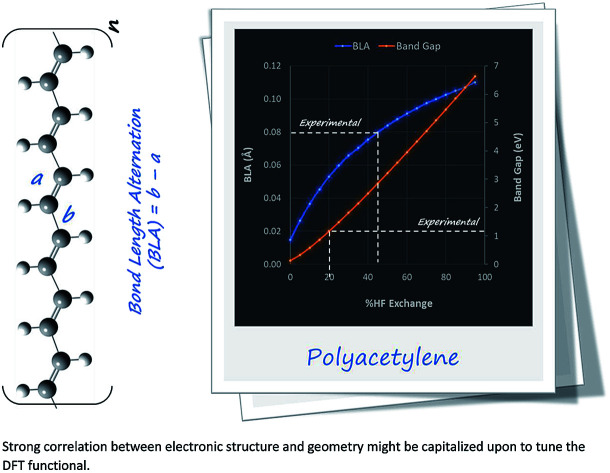

## Introduction

It would be absurd to deny the importance of quantum electronic structure theory in modern materials chemistry research. Theory provides a way to design and screen materials for desired properties, reducing the environmental impact and costs of synthesis and experimental characterization. Theory provides insight into how materials properties are determined by atomic-scale structure. Theory can even provide a “sanity check” when an experiment produces an unexpected result. Today it seems that nearly every major funded materials chemistry research program includes a theory & computation component, as do a huge fraction of the resulting publications. Of course, a great deal of this computational work is founded on the wildly popular density functional theory (DFT). Its use for the above-mentioned design and screening applications is predicated on the assumption that it accurately predicts geometric and electronic structure.

In general, DFT provides a good balance between accuracy and computational expense for materials systems. By contrast, more accurate many-body methods (*e.g.* CCSD and *GW*) are computationally expensive, prohibitively so in many cases. At the other end of the spectrum, tight binding methods are typically computationally inexpensive to employ, but may provide only a qualitative description of the electronic structure. DFT calculations address electronic interactions at the quantum level so they provide true electronic structure information, which is essential to understanding band structure, band gap, *etc.* DFT is efficient and reasonably accurate for predicting electronic properties of materials without, or with minimal, empiricism. On current computational hardware DFT is capable of routine modeling of unit cells containing 100–1000 atoms. For example, our own work applied DFT with hybrid functionals to a metal–organic hybrid graphene analogue, including defect-containing structures whose unit cell contains 750 atoms.^1^ That work demonstrates the practicality of hybrid DFT calculations on large unit cell systems.

Despite its near ubiquitous application, however, there are shortcomings associated with DFT for the prediction of materials electronic structure. A principal goal of the present paper is to offer a perspective on these shortcomings. Fundamentally, the shortcomings associated with DFT stem from lack of knowledge of the exact functional form of the exchange–correlation functional. Inaccuracies therefore arise from using an approximate functional. These inaccuracies can be reduced by judicious selection of the approximate functional because some functionals are better for estimating some properties and others for other properties (which may or may not have been by design). Other apparent shortcomings are simply manifestations of misuse or improper application of the method. These apparent shortcomings need to be recognized because they can obscure the true limitations of a functional, and of even greater consequence, lead to accurate methods being dismissed as unreliable. Specific objectives of this paper are: to highlight a few of the shortcomings of DFT for electronic structure prediction, to distinguish those that are true shortcomings of the theory from those that merely represent its misuse, and to suggest a potentially fruitful avenue of investigation by which the accuracy of DFT for electronic structure prediction might be improved.

## The challenge of predicting the band gap

The challenge of predicting the band gap of a material will serve as a touchstone for this narrative. The band gap is critical to numerous electronic and photonic properties of a material. For example: it governs the thermal dependence of charge transport in semiconducting materials. It is related to the optical properties (absorption spectrum). Bulk heterojunctions depend on the alignment of orbital energies. Solar cells need materials with ideal band gaps to efficiently harvest light, as do UV/IR photodetectors. Although one might argue that other intrinsic electronic structure properties such as conductance and/or carrier mobility are actually more relevant in practical applications, the answer to the question, “what's the gap?” serves as a simple and useful way to screen new materials for possible electronic applications. Consequently, there is a substantial demand for a theoretical tool (*i.e.* “canned” quantum electronic structure software) capable of accurate *a priori* predictions of the band gap in the hands of a trained but non-specialist user.

## An incorrect structural model can yield an incorrect band gap

As an example, consider the case of fullerite. Fullerite is the solid crystalline form of buckminsterfullerene, C_60_. As noted by Katz,^2^ the first prediction of the electronic structure of the C_60_ molecule is usually attributed to Bochvar and Gal'pern^3^ in 1973. Davidson also reported early predictions of its electronic structure, in 1981.^4^ These theoretical studies captured key qualitative features of the electronic structure, such as the large gap between the highest energy occupied molecular orbital and the lowest energy unoccupied molecular orbital (HOMO–LUMO gap) as well as the presence of high-degeneracy orbitals, but the findings were not quantitatively accurate. They also appear to have been largely ignored, only to gain recognition after the experimental discovery of C_60_ by Kroto *et al.*^5^ It's doubtful that quantitatively accurate predictions would have drawn any greater attention (Perhaps the scientific community was not ready to appreciate fullerenes.) but after the discovery of C_60_, the scientific community was very definitely interested in accurate predictions of its electronic structure. One of the first attempts to do so was reported by Saito and Oshiyama.^6,7^ Using DFT with the local density approximation (LDA) they found the HOMO–LUMO gap in isolated C_60_ in the gas phase to be 1.9 eV and the band gap in fullerite to be 1.5 eV. Given that the individual C_60_ clusters in fullerite are held together predominantly by van der Waals forces, it seems reasonable to suppose that the electronic band structure in fullerite would be closely related to the molecular orbitals of isolated C_60_,^2^ and the results of Saito and Oshiyama^6,7^ would appear to bear that out – the “LDA gap” in fullerite is quite close to that in isolated C_60_.

How accurate is the LDA prediction of the fullerite band gap? Experimental studies found the band gap to be in the range 1.8 eV (ref. 8)–2.3 eV (ref. 9). At face value, the LDA calculations do reasonably well, with the computed gas phase HOMO–LUMO gap being in slightly better agreement with experiment than the computed band gap in the crystalline solid, but there are much deeper considerations.

In determining the “band gap” (*E*_g_) one is attempting to ascertain the energy difference between the low-energy edge of the conduction band and the high-energy edge of the valence band. This is the fundamental gap. For a gas phase system it is, by definition, the difference between the first ionization energy and the electron affinity.^10^ This quantity is readily accessible from electronic structure calculations on the neutral system, its corresponding cation and anion. By Koopmans' theorem (Janak's theorem^11^ in the case of DFT) it is approximated by the HOMO–LUMO gap.

Experimentally, several strategies have been employed to obtain the gap energy. A typical approach for solids is to extrapolate the gap energy from a Kubelka–Monk plot of the diffuse reflectance spectrum (DRS). The extrapolation strategy is used to avoid biasing the estimate of *E*_g_ with localized states that are not formally considered part of the band structure of the bulk material. As an alternative strategy for a semiconducting material, an Arrhenius-style plot of the natural log of conductance (ln *Γ*) *versus* reciprocal temperature (1/*T*) will exhibit a straight line of slope equal to (
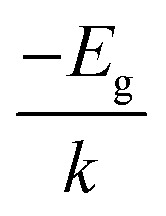
) where *k* is the Boltzmann constant, from which *E*_g_ may be extracted, assuming that *Γ* is proportional to the concentration of charge carriers and that such carriers are produced by thermal promotion of electrons from the valence band maximum (vbm) to the conduction band minimum (cbm). In a gas phase system, the gap is often inferred from the photoexcitation spectrum. In this case one is measuring the optical gap. It is the lowest energy dipole-allowed transition that starts in the ground state of the system. Because of the Coulomb interaction between the excited electron and the hole left behind upon its excitation, the optical gap is always lower in energy than the fundamental gap, the difference being due to exciton binding.^12,13^ It is therefore important to note that an accurate calculation of the fundamental gap will generally not be in quantitative agreement with an accurate measurement of the photoexcitation energy. Robust comparison must consider exciton binding, a consideration often neglected in the literature.

It is also important that experimental investigations of the band gap be carried out on pristine samples to accurately assess *E*_g_. The presence of defects can produce gap states that allow for increasing the concentration of charge carriers at lower energy cost.^14^ Additionally, defects can open a gap in an otherwise metallic material.^15^ Defects then can introduce errors into measurements of *E*_g_, but what about the accuracy of theoretical calculations?

Energy bands occur in systems of translational symmetry where they arise from linear combinations of the molecular-orbital-like contributions from a unit cell and all of its translational images. In an infinite periodic array, the fully symmetric combination of an orbital and all of its images marks one energy edge of a band and the fully antisymmetric combination marks the other energy edge.^16^ All other linear combinations fall between these energy extremes, effectively producing a continuous curve in energy–momentum space. The stronger the interactions among the constituent orbitals in adjacent cells, the larger the range of energies spanned by the band. This span of energies is termed the dispersion of the band.

When a small and finite (molecular-scale) model of a bulk material is used, no longer are there an effectively infinite number of translational images of the fundamental unit. Consequently, only a small finite number of independent linear combinations of the constituent orbitals are possible and energy space between the fully symmetric and fully antisymmetric combinations is not filled but contains only a small finite number of discrete orbital energies. The energy gap between the LUMO and the HOMO is often taken to approximate the band gap in the bulk material. This approximation breaks down in at least two situations: (1) the constituent orbitals interact strongly so that the energy bands in the solid are highly dispersive. (2) The cluster model is small so that very few orbitals exist within the energy space spanned by the corresponding band of the bulk material; in other words, the model does not represent the bulk material. The role of dispersive bands and the consequences of using a finite cluster model are extremely important considerations in comparisons of experimentally determined band gaps to theoretically computed ones.

Let us now return to the example of C_60_. Any macroscopic sample of C_60 (s)_ is likely to be, or closely resemble, fullerite. Consequently, band gap determination by extrapolation of diffuse reflectance spectra, or extraction from an Arrhenius plot of (ln *Γ*) *versus* (1/*T*) is probing the band structure of the solid, not the molecular orbitals of an isolated molecule. Estimates of *E*_g_ of a bulk material based on the HOMO–LUMO gap of isolated C_60_ are therefore inherently flawed. Computations of the HOMO–LUMO gap in isolated C_60_ can therefore be forgiven if they fail to reproduce the experimental band gap in fullerite; they are based on a model of a physically different system. (Whether one should forgive a scientist for employing such an approximation is an entirely different matter.) Perhaps surprisingly, the use of the HOMO–LUMO gap in a finite cluster model as an estimate of *E*_g_ is widespread. Assorted theoretical estimates of the band gap in fullerite and C_60 (g)_ are collected in Table 1, which includes numerous such cases.

**Table 1:** Collection of experimental and computational values of the band gap (fullerite) and HOMO–LUMO gap (C_60_)

System	Geometry method	*E*_g_ method	*E*_g_ (eV)	Reference
**Theoretical results**				
Fullerite	LDA-OPT	LDA	1.04	17
Fullerite	LDA-OPT	GW	2.15	17
Fullerite	OPT	PBE	1.091	18
C_60_	Expt.	LDA	1.9	6 and 7
Fullerite	Expt.	LDA	1.5	6 and 7
C_60_	Not specified	TB	3.01	19
C_60_	B3LYP-OPT	B3LYP	2.743	20
C_60_	B3LYP-OPT	PBE	1.65	20
C_60_	OPT	PBE	1.668	21
Fullerite	OPT	LDA	1.18	22
C_60_	Not specified	LDA	1.58	23
C_60_	OPT	B3LYP	1.9	24
(C_60_)_6_	OPT	B3LYP	1.8	24
C_60_	Not specified	GW	4.91	23
Fullerite	Expt.^25^	HSE06	1.8	Present work
C_60_	Expt.	B3LYP	2.742	Present work
C_60_	Expt.	LC(*μ*_opt_ = 0.197)-PBE/6-311G(d,p)	5.4	Present work

**Experimental results**				
C_60_	—		4.9	23
C_60_	—		4.66	26
Fullerite	—		1.86 ± 0.1	27
Fullerite	—		2.3 ± 0.1	9

The fact that LDA calculations of the HOMO–LUMO gap in gas phase C_60_ are in better agreement with the band gap in fullerite than LDA calculations of the band gap of the full periodic solid must be interpreted in light of the structural models upon which they are based. LDA/C_60 (g)_ is not a better model of the electronic structure of fullerite. The agreement is serendipitously better. The HOMO–LUMO gap is an approximation of its photoexcitation energy. The best available experimental data show this fundamental gap to be 4.66–4.9 eV,^23,26^ which exceeds the LDA estimate quite considerably. LDA/C_60 (s)_ more closely resembles that of fullerite. The best available experimental data show the band gap of fullerite to be 1.86–2.3 eV,^9,27^ which exceeds the LDA estimate, 1.04 eV, quite considerably. In fact, it is very well-known that LDA tends to underestimate energy gaps between occupied and unoccupied energy levels.^28^ The LDA/C_60 (g)_ HOMO–LUMO gap is closer to the experimental band gap in fullerite only because it is severely underestimating a much larger value.

The principal take-away from the above discourse is that before dismissing a theoretical method as inaccurate, or accepting it as accurate and reliable for a given system, one should be sure that the theoretical method is being applied to an appropriate structural model of the system being subjected to experimental characterization. Finite gas-phase systems are not appropriate models of (effectively) infinite periodic solids, even if the computed band gap appears to be in better agreement with experimental results.

## An inappropriate functional can yield an incorrect band gap

Some erroneous theoretical predictions of *E*_g_ can be attributed to an incorrect structural model, but the data in Table 1 show clearly that DFT with the LDA functional (and some other functionals) fails even when an appropriate structural model is employed. Of course this is a widely known failure of LDA; it tends to underestimate *E*_g_ in gapped systems.^28^ Fortunately, a plethora or alternative functionals exist (many are standard options in commercial electronic structure software) but selecting from these is an art at best, and often seems to be little more than guesswork. Let us consider why this is so, and what can be done to address the problem.

As alluded to above, within the DFT formalism, although the true exact exchange–correlation functional (*V*_xc_) is unknown, there is freedom to select from a wide variety of approximate exchange–correlation functionals.^29^ Traditional local functionals (*e.g.* LDA wherein *V*_xc_ is assumed to depend on the local electron density, and GGA wherein *V*_xc_ is assumed to depend on the local density with a correction that depends on the derivative of the local density) work well for metallic systems, but over delocalize the electron density, leading to an underestimate of the band gap in gapped systems. By contrast, when Hartree–Fock exchange (HF-ex) is used in the absence of accurate treatment of correlation effects (which partially screen HF-ex), the electron density is over localized, which leads to an overestimate the of band gap in gapped systems and complete failure in metallic systems. Traditional hybrid functionals attempt to find a compromise between the local functionals and full HF-ex by incorporating a fraction of the HF-ex. This compromise approach is not universally successful because the contribution from HF-ex is somewhat distance-dependent. An effective strategy to deal with these competing strengths and weaknesses is to develop range-separated density functionals such as long-range corrected functionals and screened-hybrid functionals. In these functionals the inter-electron Coulomb operator is split into two (or more) terms each active over a different length scale.^29^ HF exchange is therefore explicitly modulated as a function of electron–electron distance instead of simply being weighted by a global constant (as is the case with traditional hybrid functionals). A fraction of the exchange contribution is taken to be HF-ex, but a sigmoid function is used to “switch off” the HF-ex contribution at appropriate length scales. The range separation parameters control the switching point(s).

In the long-range corrected (LC) strategy, 100% HF-ex is used at long range but it is attenuated at short range. This approach produces an effective potential with the correct asymptotic behavior.^29^ For molecular systems it improves accuracy in the prediction of molecular orbital energies, and hence also excitation energies.^30^ This improvement is a result of getting the correct energy curvature with respect to the number of electrons.^31,32^

The screened hybrid strategy has been especially effective for the treatment of periodic systems. In extended systems, HF-ex is naturally screened at long range. Moreover, calculation of HF-ex at long range would be very expensive, DFT is cheaper. To address both issues, DFT is used for all length scales and even though it doesn't capture all of the exchange, that's ok at long range because exchange is screened at long range anyway. At short range, since DFT is missing some of the exchange and one can't dismiss the remainder due to screening, a fraction of HF-ex is added in. Presumably the strategy is to try to add-in just enough HF-ex to compensate for the exchange that is missing from DFT. Since the range over which HF-exchange is computed is limited, the expense of computing HF-ex is controlled.

While numerous screened functionals are in wide use, there is no widely accepted procedure for matching the functional to the problem at hand. Functionals may accurately predict geometric structure yet give poor predictions of the band gap, or *vs.* versa. It has been found, for example, that the HSE06 (ref. 33 and 34) functional often more accurately predicts the band gap for solid materials when the electronic structure is simply computed using an experimental geometry than if the structure is first optimized with DFT.^35^ In fact, we will show below that HSE06 provides a surprisingly reliable prediction of the band gap in solids when reliable structural information is available, with two allotropes of elemental phosphorus, and polyacetylene being used as case studies.

For gas-phase structures/molecules, a variety of techniques produce a reliable HOMO–LUMO gap given the experimental geometry. A “single shot” *G*_0_*W*_0_ calculation, if computationally feasible, is typically reliable for predicting quasiparticle properties. Note, for example, the excellent accuracy of *G*_0_*W*_0_ in predicting the HOMO–LUMO gap in C_60_ (see Table 1). Within the LC-DFT formalism, non-empirically tuned range-separated DFT has been shown to produce fundamental gaps in good accord with corresponding predictions from *G*_0_*W*_0_ for DNA/RN nucleobases^10^ and accurate orbital energies and electron affinities in atomic anions.^36^ In the absence of LC functionals, DFT with the B3LYP functional is generally much more reliable for HOMO–LUMO gap predictions than calculations using LDA or any of various gradient-corrected functionals.

**Fig. 1:**
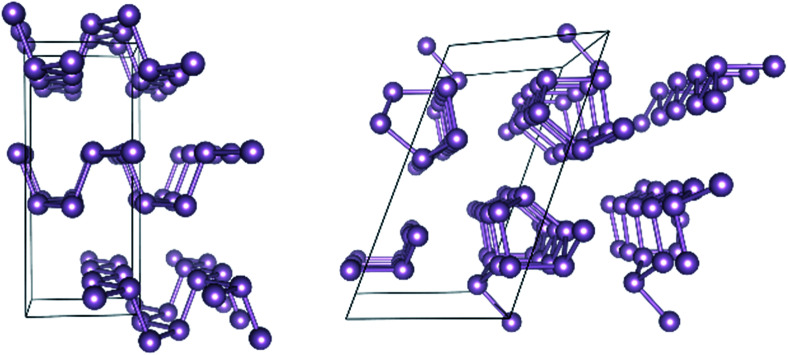
Geometric structure of black phosphorus (left) and red phosphorus (right).

Turning next to solid state structures, the case of elemental phosphorus will serve as a representative example. Phosphorus has received considerable attention recently because it forms layered structures reminiscent of graphite. There has been much speculation that few-layer phosphorus might share the features of graphene that render the latter attractive for microelectronic applications, while also still exhibiting the semiconducting property absent from graphene that renders it inappropriate for such applications.^37^ Two allotropes of elemental phosphorus are especially promising, black phosphorus (BP) and red phosphorus (RP). Their structures are shown in Fig. 1.

BP has a band gap of 0.33 eV.^38^ When the structure is optimized at the GGA-D2 level of theory, the predicted band gap is 0.25 eV,^39^ a result consistent with the well-known underestimate of the band gap by GGA. We performed an HSE06 calculation on the experimental crystal structure and found the predicted gap to be 0.37 eV, in excellent agreement with experiment (see Table 2).

**Table 2:** Collection of experimental and computational values of the band gap in BR & RP

System	Geometry method	*E*_g_ method	*E*_g_ (eV)	Reference
**Theoretical results**				
BP bulk	PBE-D3	PBE-D3	0.37	40
4L BP	PBE	PBE	0.18	41
1L BP	PBE	HSE06	1.00	42
BP bulk	PBE	HSE06	0.31	42
BP bulk	optB88vdW	HSE06	0.35	43
1L BP	optB88vdW	HSE06	1.51	43
2L BP	optB88vdW	HSE06	1.02	43
3L BP	optB88vdW	HSE06	0.79	43
4L BP	optB88vdW	HSE06	0.65	43
5L BP	optB88vdW	HSE06	0.59	43
BP bulk	GGA-D2	GGA-D2	0.25	39
1L BP	Expt. crystal	*G*_0_ *W*_0_	2.0	44
2L BP	Expt. crystal	*G*_0_ *W*_0_	1.3	44
3L BP	Expt. crystal	*G*_0_ *W*_0_	1.1	44
BP bulk	Expt. crystal	*G*_0_ *W*_0_	0.3	44
2L BP	Expt. crystal	HSE06	1.06	Present work
3L BP	Expt. crystal	HSE06	0.86	Present work
BP bulk	Expt. crystal	HSE06	0.37	Present work
RP bulk	Expt. crystal	PBE-D2	1.50	45
RP bulk	Expt. crystal	HSE06	1.97	Present work

**Experimental results**				
BP bulk	—	IR	0.3	46
1L BP	—	Photoluminescence	1.75	47
2L BP	—	Photoluminescence	1.29	47
3L BP	—	Photoluminescence	0.97	47
4L BP	—	Photoluminescence	0.84	47
5L BP	—	Photoluminescence	0.8	47
BP bulk	—	Photoluminescence	0.295	47
BP bulk	—	*Γ*(*T*)	0.33	38
RP bulk		UV/vis	1.9	48
RP bulk		UV/vis	2.02	49

Next consider few-layer BP. Experimental and theoretical band gap estimates are collected in Table 2. Again, note that PBE severely underestimates the band gap. Qiao *et al.*^43^ found that they could do better by first optimizing the structure using the optB88vdw functional, then performing a HSE06 single-point calculation to estimate the band gap. We have estimated the band gap with single point calculations carried out on the experimental crystal structure^50^ and found even slightly better agreement with experiment (see Table 2). In fact, for both bulk and few-layer BP, HSE06 calculations on the experimental crystal structure produce band gap estimates nearly as accurate as those from *G*_0_*W*_0_ calculations.

The second allotrope of phosphorus that has received attention for its layered structure is RP.^51^ Collected in Table 2 are experimental and theoretical values for the band gap in RP. The story mirrors that for BP. PBE calculations are inaccurate, underestimating the gap. Recently the crystal structure of RP has been reported.^51^ We have carried out an HSE06 single point calculation using this structure and found a predicted band gap of 1.97 eV, in excellent agreement with experimental values of 1.9 eV due to Roshith^48^ and 2.12 eV due to Ji.^52^

The above results for fullerite and elemental phosphorus suggest that HSE06 is a reliable functional for predicting band gap in solid materials, if it does not need to be relied upon to first predict the structure. Our insight is the recognition that the interplay between the geometric and electronic structure might be used to optimize the theory/functional: in a reliable theoretical method, accurate prediction of the electronic structure is an indication that the predicted geometry is correct. If we know that an admixture of DFT and HF exchange will accurately describe the electronic structure of material systems, the question becomes, what mixture? Knowing that some mixture is correct may allow us to find the correct mixture using structure prediction.

## A strategy to improve DFT predictions of band gap

### Conjugated polymers

As an example, we have considered the structure and electronic properties of polyacetylene. Polyacetylene is a semiconducting conjugated polymer, *i.e.* it has alternating single and double bonds. Experimentally, the band gap is 1.2 eV and the bond length alternation (BLA) is 0.08 Å. Table 3 shows the predicted values of these quantities as determined with several well-known DFT functionals.

**Table 3:** Band gap and bond length alternation in polyacetylene as predicted with several popular DFT functionals

	Band gap (eV)	BLA (Å)
HF	7.2	0.123
PBEh (PBE0)	1.5	0.060
HSE06	0.8	0.050
PBE	0.1	0.015

**Fig. 2:**
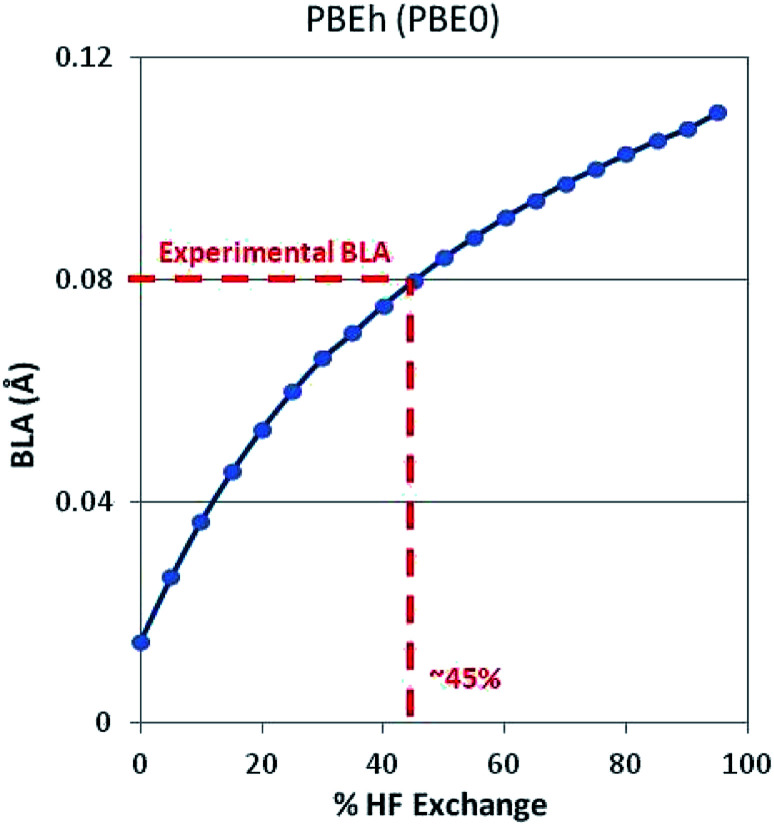
BLA in polyacetylene as predicted with DFT-PBEh for varying degrees of HF exchange. Note that at 45% HF exchange, the exact experimental BLA is recovered. When this structure is used as input to a DFT-HSE06 SP calculation of polyacetylene, the correct (experimentally observed) band gap, *E*_g_ = 1.2 eV, is predicted.

Note that there is strong correlation between band gap and BLA. As might be expected, Hartree–Fock theory over localizes the electron density causing a large BLA and large bandgap. (Electron density is heavily localized in the double bonds and the barrier to electron movement is too large.) GGA-DFT (PBE) over delocalizes the electron density causing a small BLA and small bandgap. (Electrons can move too easily.) Significant improvements are achieved by adding a faction of HF exchange to DFT such as with the PBEh (a.k.a. PBE0) functional, which incorporates 25% HF exchange. This is a manifestation of the dependence of BLA on the degree to which HF-ex is included in both global and long-range-corrected hybrid functionals.^53,54^ Extending this concept, we optimized a 1D periodic model of polyacetylene using DFT and a PBEh-style functional with varying degrees of HF exchange. As shown in Fig. 2, when using 45% HF exchange, DFT correctly predicts the experimentally observed BLA. This strategy works, in part, because intramolecular interactions are strongly dependent on %HF exchange.

**Fig. 3:**
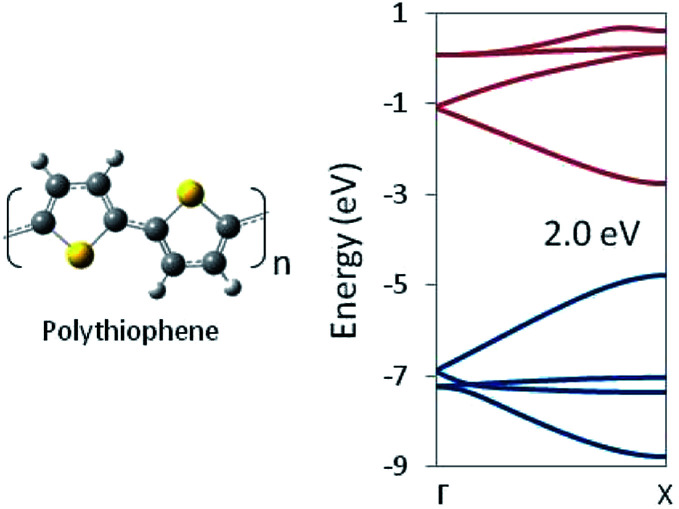
Polythiophene monomer unit and G → X band structure as computed at the HSE06/PBEh(45% HF) level of theory. The band gap is in quantitative agreement with the experimental value.

In this way, structural information may be used to tune the DFT functional. Thus tuned, that functional can then be used to optimize chemically related structures for which no experimental geometry is available. Structures optimized with the tuned functional may be used in-turn as input for an HSE06 (or other appropriate functional) calculation of the electronic structure and trusted to provide a reliable prediction of the band gap. For example, we employed our optimized PBEh(45% HF) functional with no further adjustment to a 1D periodic model of polythiophene. The predicted HSE06 band structure is shown in Fig. 3, which shows a band gap of 2.0 eV, in essentially exact agreement with experimental measurements, despite the fact that no quantitative experimental geometry was available for the system. Note that polythiophene involves S atoms whereas polyethylene does not, yet tuning the DFT functional to the latter, produces a functional that gets both the geometric and electronic structure of the former correct, suggesting a degree of transferability in the method.

### Conjugated molecular systems

**Fig. 4:**
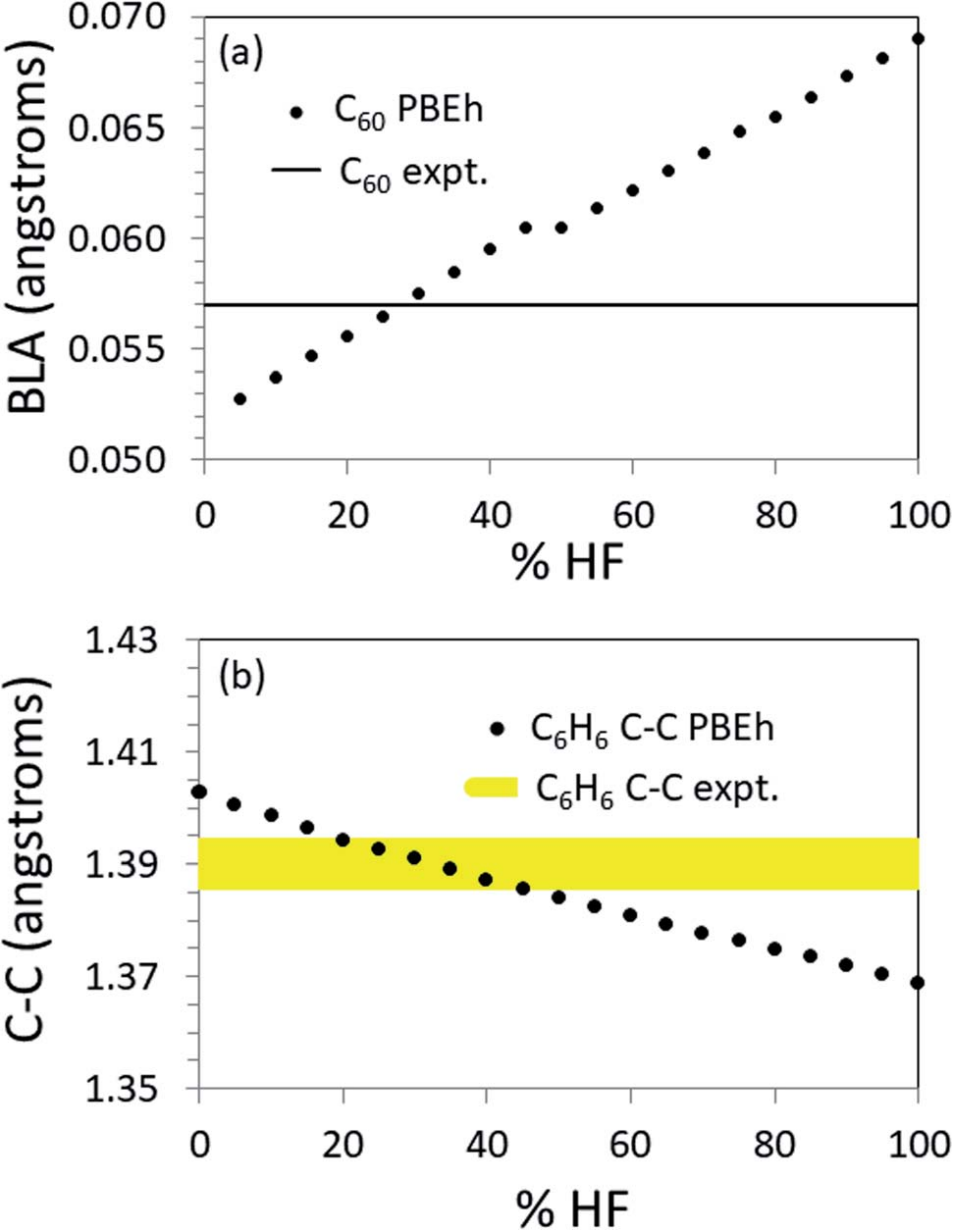
Structural parameters as a function of %HF when calculated with PBEh. Top: BLA in C_60_. Note that the experimental BLA determines the optimum %HF quite tightly. Bottom: Bond length in benzene. Note that the range [20 ≤ %HF ≤ 45] produces the correct bond length to within ±0.005 Å, as shown by the yellow band.

To further explore the transferability of the approach, we carried out calculations on several finite systems with extended conjugation akin to that in polyacetylene. Fig. 4 shows the values of key structural parameters related to the π-conjugation as a function of %HF exchange used in the PBEh functional for propene, benzene and C_60_. (For more complete numerical results, see the ESI.†)

For C_60_, the BLA is most accurately reproduced by PBEh with 25–30% HF exchange (see Fig. 4a). Note that using this %HF exchange, the predicted short and long bonds are in error by just 0.7% (see ESI†). When the structure is known to this level of accuracy, methods exist that will accurately predict the HOMO–LUMO gap as shown in Table 1. As another example, given an accurate structure, non-empirically tuned range-separated DFT accurately predicts the band gap in PCBM,^55^ a functionalized C_60_.

For benzene there is no BLA. The experimental bond length is 1.39 Å. PBEh calculations in the range [20 ≤ %HF ≤ 45] produce the correct bond length to within ±0.005 Å (see Fig. 4b). Again, at this level of structural accuracy, electronic structure methods exist that will accurately predict the HOMO–LUMO gap. Interestingly, by interpolation, 22% HF produces a HOMO–LUMO gap in the best agreement with the UV/vis absorption maximum when PBEh is used. This is very close to the default %HF in PBE0 and the in the popular B3LYP functional, perhaps indicative of the presence of benzene in the training set.

For propene, the “best” %HF depends on whether one considers the length of the short (double) bond, the length of the long (single) bond, or the BLA as the guiding structural parameter (see ESI†). No UV/vis absorption data is available, but a large-basis CCSD(T) calculation^56^ predicts a HOMO–LUMO gap of 6.8 eV, which would correspond to 12% HF when using PBEh.

It is perhaps unsurprising that the standard PBEh functional contains 25% HF exchange, as this is close to an average of the “best” value seen in the above sample of finite systems. From this study of the above set of three model systems, however, it is clear that no single fixed %HF works in all cases. A more ideal situation would be to non-empirically tune the %HF for the system under consideration. These results demonstrate that structural properties are %HF dependent; therefore, it could play a leading role in the objective function for a robust tuning approach.

## Prospects

This method of optimizing the DFT functional proposed here is an innovation that has the potential to offer the capability to perform accurate predictions of electronic structure without precise quantitative X-ray structural models. At least three cases merit consideration:

In the first case, accurate information about only a single structural parameter that is highly %HF dependent need be available. BLA in conjugated systems is a representative example. The %HF is then adjusted so that this parameter is accurately predicted. The overall structure is then optimized with PBEh using this %HF exchange and a subsequent SP calculation carried out for band gap prediction, screened-hybrid functional in the case of an extended solid and a non-empirically tuned LC functional in the case of a molecular system.

In the second case, accurate spectroscopic information is available, but only a qualitative structural model (*i.e.* correct connectivity information). In principle, the structure can then be reverse-engineered by applying an electronic structure model that is known to deliver accurate electronic structure information based on an accurate structure. For example, one could use HSE06 in the case of an extended solid and a non-empirically tuned LC-DFT calculation in the case of a molecular system. The geometric structure is systematically varied and the electronic structure computed for each geometry until a geometry is identified that correctly predicts the accurately known features of the electronic structure. In practice such a procedure could be very tedious and would lead to good structural accuracy only when the HOMO–LUMO gap shows strong structure dependence.

In the third case, only a qualitative structural model is available (*i.e.* correct connectivity information). The structure is first optimized using a DFT functional that has been tailored for chemically similar systems for which detailed geometric structure information is available. The band structure at the optimized geometry is then predicted with a SP calculation using a technique well-vetted for electronic structure predictions, again a screened-hybrid functional in the case of an extended solid and a non-empirically tuned LC functional in the case of a molecular system. This is the scenario in which a procedure for non-empirical tuning of the %HF for accurate structure prediction would be most valuable.

The above discussion outlines a potentially reliable strategy for tuning the DFT exchange–correlation functional, but it is contingent on the availability of sufficiently good experimental data for the system of interest or a relevant related system. The acquisition and tabulation of high accuracy structural data is therefore a critical need for the theory community. High accuracy structural data for a wide variety of materials systems is needed to develop methods for accurate *a priori* structure prediction, and for subsequent implementation of those methods into widely available “canned” quantum electronic structure software programs that are accessible to non-specialist users.

## Conclusions

There is substantial demand for theoretical/computational tools that can produce correct predictions of the geometric structure and band gap to accelerate the design and screening of new materials with desirable electronic properties. DFT-based methods exist that reliably predict electronic structure given the correct geometry. For example, tuned long-range corrected hybrid functionals work well to obtain an accurate prediction of the electronic structure in solid systems. An example is the HSE06 functional. When good structural data are available, HSE06 can often be trusted to generate a reliable description of the electronic structure. When good spectroscopic data is available, it could, in principle, be used as input to the inverse problem of generating a good structural model. For gas-phase systems, a non-empirically tuned LC functional, where the HOMO(LUMO) energy is matched to the IP(EA), yields *G*_0_*W*_0_-quality quasiparticle energies given good geometric structure data is employed.

Herein we have argued that when neither structural data nor spectroscopic data is available, but good structural data is available for a chemically related system, the strong correlation between electronic structure and geometry might be capitalized upon to tune the DFT functional. The tuned functional could then be used in-turn to determine the structure of the system of interest with sufficient accuracy that a subsequent HSE06 calculation (solid) or non-empirically tuned LC-DFT calculation with a range-separated functional (molecular system) will yield a reliable band structure. The procedure is demonstrated for polyacetylene/polythiophene and for C_60_. This strategy appears to bear sufficient promise to warrant further development.

There are numerous systems of considerable current interest for which adequate structural data are not available. A common situation is to have accurate lattice constants for a crystalline material, and to know the connectivity of the constituent atoms, but not their exact coordinates. The ability to tune a DFT functional for accurate structural predictions, would then offer the opportunity to obtain quality electronic-structure predictions as well. The availability of high-accuracy structure data would serve as a driver to advance the field. We therefore conclude this narrative with an appeal for new high-accuracy characterization efforts. Good experimental data will drive theory forward. We further appeal to theorists to pursue non-empirical tuning procedures for accurate geometric structure predictions, because given a good structure, theoretical methods exist that are computationally tractable and can answer the question, “What's the gap?”

## Conflicts of interest

There are no conflicts of interest to declare.

## Supplementary Material

RA-010-D0RA07496A-s001
