# Supplementary material for: What's the gap? A possible strategy for advancing theory, and an appeal for experimental structure data to drive that advance
Source: RSC Adv. 2020 Oct 6;10(60):36887–96. doi: 10.1039/d0ra07496a (PMC9057033; doi:10.1039/d0ra07496a)
Supplement: RA-010-D0RA07496A-s001 [file RA-010-D0RA07496A-s001.pdf]

Electronic Supplementary Material (ESI) for RSC Advances.
This journal is © The Royal Society of Chemistry 2020

Supplementary Information to accompany:

What's the gap? A possible strategy for advancing theory, and an appeal for experimental structure data to drive that advance.

Karl Sohlberg†‡* and Mike Foster†*,

Department of Chemistry and Department of Materials Science & Engineering, Drexel University, Philadelphia, PA, 19104

†Department of Chemistry, Drexel University, Philadelphia, PA, 19104

‡Department of Materials Science & Engineering, Drexel University, Philadelphia, PA, 19104

*Authors for correspondence

C60					Benzene		Propene			
%HF	C=C	C-C	BLA	BG	C…C	BG	C=C	C-C	BLA	BG
5	1.4018	1.4546	0.0528	1.936	1.4006	5.603	1.3393	1.4998	0.1605	6.138
10	1.3990	1.4527	0.0537	2.207	1.39872	6.013	1.3370	1.4983	0.1613	6.613
15	1.3962	1.4509	0.0547	2.481	1.39650	6.425	1.3349	1.4970	0.1621	7.087
20	1.3935	1.4491	0.0556	2.757	1.39432	6.837	1.3328	1.4957	0.1629	7.561
25	1.3909	1.4474	0.0565	3.035	1.39272	7.243	1.3308	1.4943	0.1635	8.035
30	1.3883	1.4458	0.0575	3.315	1.39088	7.652	1.3288	1.493	0.1642	8.509
35	1.3857	1.4442	0.0585	3.598	1.38908	8.059	1.3268	1.4916	0.1648	8.982
40	1.3832	1.4427	0.0595	3.882	1.38732	8.466	1.3249	1.4903	0.1654	9.456
45	1.3807	1.4412	0.0605	4.169	1.38562	8.872	1.3231	1.4890	0.1659	9.929
50	1.3789	1.4394	0.0605	4.449	1.38398	9.277	1.3213	1.4877	0.1664	10.401
55	1.3766	1.4380	0.0614	4.738	1.38238	9.682	1.3195	1.4864	0.1669	10.873
60	1.3744	1.4366	0.0622	5.028	1.38078	10.085	1.3178	1.4851	0.1673	11.345
65	1.3722	1.4353	0.0631	5.321	1.37924	10.489	1.3161	1.4839	0.1678	11.817
70	1.3700	1.4339	0.0639	5.614	1.37772	10.891	1.3144	1.4827	0.1683	12.288
75	1.3679	1.4327	0.0648	5.910	1.37628	11.293	1.3128	1.4814	0.1686	12.759
80	1.3659	1.4314	0.0655	6.207	1.37482	11.694	1.3112	1.4802	0.1690	13.229
85	1.3638	1.4302	0.0664	6.505	1.37342	12.094	1.3097	1.4790	0.1693	13.700
90	1.3618	1.4291	0.0673	6.806	1.37208	12.494	1.3081	1.4779	0.1698	14.170
95	1.3599	1.4280	0.0681	7.108	1.37028	12.899	1.3067	1.4768	0.1701	14.639
expt	1.401[1]	1.458[1]	0.057	4.66…4.9[2,3]	1.39[4]	6.966[4]	1.353[4]	1.488[4]	0.135[4]	6.8[4]*
best %HF	10	0	27.5	55	20-45	22	0	45	0	12

Table S1. HOMO-LUMO gap and various structural parameters in C_{60}, benzene and propene as a function of %HF exchange used in PBEh. Results are reported to several digits of precision for comparison purposes. The number of digits is not intended to imply computational accuracy. *denotes theoretical CCSD(T) value in lieu of expt. data.

References for SI:

1 Hedberg, K. *et al.* Bond lengths in free molecules of buckminsterfullerene, C60, from gas-phase electron diffraction. *Science* **254**, 410-412 (1991).

2 Wang, X.-B., Ding, C.-F. & Wang, L.-S. High resolution photoelectron spectroscopy of C_{60}. *The Journal of Chemical Physics* **110**, 8217-8220, doi:10.1063/1.478732 (1999).

3 Blase, X., Attaccalite, C. & Olevano, V. First-principles *GW* calculations for fullerenes, porphyrins, phtalocyanine, and other molecules of interest for organic photovoltaic applications. *Physical Review B* **83**, 115103, doi:10.1103/PhysRevB.83.115103 (2011).

4 Johnson, R. D. I. Vol. Number 101 (NIST Standard Reference Database, 2019).
